# Do-Nothing Prebiotic Chemistry: Chemical Kinetics as a Window into Prebiotic Plausibility

**DOI:** 10.1021/acs.accounts.4c00247

**Published:** 2024-12-19

**Authors:** Skyla B. White, Paul B. Rimmer

**Affiliations:** Astrophysics Group, Cavendish Laboratory, University of Cambridge, JJ Thomson Avenue, Cambridge CB3 0HE, United Kingdom

## Abstract

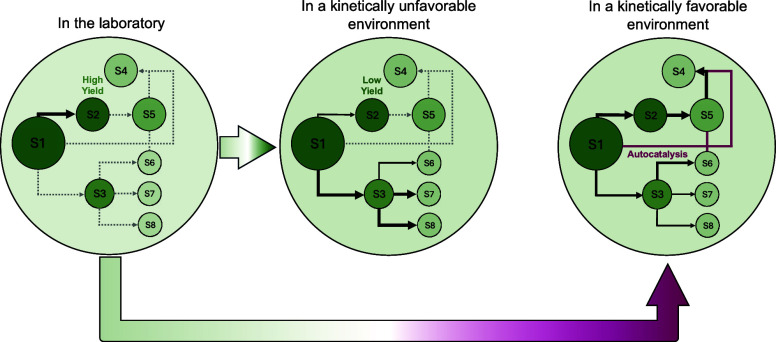

Origin of Life research is a fast growing field of study with each year bringing new breakthroughs. Recent discoveries include novel syntheses of life’s building blocks, mechanisms of activation and interaction between molecules, and newly identified environments that provide promising conditions for these syntheses and mechanisms. Even with these new findings, firmly grounded in rigorous laboratory experiments, researchers often find themselves uncertain about how to apply them. How can a bridge be built between the laboratory and the geochemical environment? A critical question to ask when seeking to apply new results in origins is: how can this chemistry occur without direct intervention from a chemist? We believe the first step toward answering this question lies in the determination of rate constants and the construction of chemical networks to describe prebiotic chemistry in geochemical environments.

So far, our group has measured several rate constants relevant to different prebiotic reaction networks, starting with the synthetic pathways of the cyanosulfidic network. The reactions we explore often involve ultraviolet light-driven photochemistry, facilitated by our StarLab setup that accurately simulates the spectrum of the young Sun and other stars. Our latest work investigates environments with active photochemistry in the absence of cyanide. In this study, we measure the effective rate constant for the production of formate from the reduction of carbon species using sulfite within the context of early Martian waters. The underlying goal of the work done in our group is to predict the likelihood that certain geological conditions will result in a specific set of chemical products. These predictions can be combined with those we have made for the necessary astrophysical conditions in certain origins of life scenarios on extrasolar planets.

In the near future we expect that a sufficient number of rate constants will be measured, by our group and others, to allow for aspects of prebiotic chemistry to be predicted using chemical kinetics models. Once these models have been benchmarked against experimental data, our next step will be applying them to natural environments that better mimic the conditions thought to have been present at the onset of life. Following this, we can test these models by comparing their predictions to additional experiments. After refinement, these models will be able to provide guidance on the optimal conditions for conducting laboratory experiments, while helping to minimize and characterize any interference from a chemist.

This approach can provide valuable insights into what is possible within geochemical environments, where all chemistry is by necessity do-nothing chemistry.

## Key References

- RimmerP. B.; XuJ.; ThompsonS. J.; GillenE.; SutherlandJ. D.; QuelozD.The origin of RNA precursors on exoplanets. Science Advances2018, 4, eaar330230083602 10.1126/sciadv.aar3302PMC6070314.^1^ This work provides estimates on how much light is needed for UV photochemistry by measuring rate constants for bimolecular reactions in the presence and absence of ultraviolet light.
- RimmerP. B.; ThompsonS. J.; XuJ.; RussellD. A.; GreenN. J.; RitsonD. J.; SutherlandJ. D.; QuelozD. P.Timescales for Prebiotic Photochemistry Under Realistic Surface Ultraviolet Conditions. Astrobiology2021, 21, 1099–112034152196 10.1089/ast.2020.2335PMC8570677.^2^ This work investigates whether the ultraviolet flux available on the surface of early Earth could facilitate a variety of prebiotic chemical syntheses.
- JiangC. Z.; RimmerP. B.; LozanoG. G.; ToscaN. J.; KufnerC. L.; SasselovD. D.; ThompsonS. J.Iron–sulfur chemistry can explain the ultraviolet absorber in the clouds of Venus. Science Advances2024, 10, eadg882638170780 10.1126/sciadv.adg8826PMC10776003.^3^ This work reports the measurement of the kinetic and thermodynamic stability of minerals in sulfuric acid, as well as the ultraviolet absorption properties of these minerals which are predicted to be present within the clouds of Venus.
- WhiteS. B.; RimmerP. B.; LiuZ.Shedding Light on the Kinetics of the Carboxysulfitic Scenario. ACS Earth and Space Chemistry2024, 8, 2133.^4^ This work describes the measurement of rate constants as a function of pH for the photochemical reduction of carbon species using sulfite.

## Introduction

The origin of life is arguably one of the greatest open problems in science and, as such, has gained a lot of traction as a formidable yet promising area of research. The motivation behind this surge in popularity is driven by recent technological advancements and new laboratory techniques that are allowing scientists to explore the intricate molecular processes and diverse environments proposed to be involved in life’s emergence.^5,6^ In addition, progress in space exploration has presented the opportunity to investigate the possibility of life beyond Earth, providing a more comprehensive understanding of the conditions that might influence the emergence of life.^7^ Despite recent advances in framing the question of life’s origins^8^ and the rapid progress in understanding the nature of prebiotic chemistry,^9^ it is difficult to assess whether we are any closer to answering the fundamental question of how life can emerge from nonliving matter.^10−12^

The question of how life began now demands a broader interdisciplinary perspective,^13,14^ making the lack of cohesion between disciplines a major barrier to advancing research within this field.^13^ Given the dearth of data and inherent uncertainties that presently inundate this work, new and old ideas, from a variety of different fields, should be considered with epistemic humility, an awareness of the limits of our knowledge.^15^ Adopting this stance will pave the way for exploring new, solvable, questions and avenues of inquiry.

To maximize the chance of discovering one or more scenarios that may lead to life’s origins, we propose that the community should focus especially on those scenarios that show the greatest empirical support. These scenarios in particular should be the most intensely tested and challenged, in order to identify and explicate experimentally and observationally grounded candidate solutions to the problem of life’s origins.^14^

This approach often begins by identifying what is inevitable in a planetary environment, or, to borrow the phrase of Benner, what “can’t not happen”.^16^ It can also start by considering what is impossible in a planetary environment, what “can’t happen”.^17^ However, between what is necessary and what is impossible, there exists what is possible yet not guaranteed.^18,19^

Often discussions of possibility can become conflated with plausibility. Prebiotic plausibility is typically applied to the chemical and physical boundary conditions of a proposed environment for prebiotic chemistry.^20−23^ Beyond boundary conditions, a complete account of prebiotic plausibility must consider the likelihood of reactions occurring in sequence under environmental conditions.^21^ We propose that these probabilities can be summarized as the translation of a chemical sequence from the laboratory to a natural environment.^19^ We further propose that these probabilities can be quantitatively estimated using kinetics.

This is the logic behind the work our lab is focused on. We begin by taking known prebiotic scenarios that have proven successful in the lab and identifying natural environments in which these scenarios could occur. We then test these reactions under prebiotically plausible conditions either with no interference from a chemist or with the interference of the chemist minimized and carefully accounted for. We term this approach do-nothing prebiotic chemistry.

Our account starts by defining do-nothing chemistry and explaining what this methodological approach has to do with the origin of life. We then introduce the analogy between do-nothing farming and do-nothing prebiotic chemistry and discuss how this do-nothing prebiotic chemistry approach can be implemented. In light of this we outline the work our lab is currently focused on in this regard, and set out goals for future work and collaborations.

## Do-Nothing Farming and Do-Nothing Prebiotic Chemistry

There is a fruitful analogy between synthetic chemistry and farming. In order to farm successfully, farmers are required to grow crops at high yields which requires fertile soil and careful cultivation. In a similar way, the synthetic chemist can selectively produce a particular chemical at high yield under certain carefully cultivated conditions. For multistep syntheses, each individual reaction needs to produce the desired product at high yield. This product may then require isolation before proceeding with the next step in the reaction sequence. Synthetic chemists who do not carry out total synthesis in this manner may encounter challenges posed by what is often referred to as the arithmetic demon.^24^ This is the idea that, if a reaction takes place over many steps, and the production rate for desired products is smaller than the rate for the competing reactions, the concentration of the desired products will drop exponentially. This idea has previously been used to place constraints on the geochemical environments where a specific prebiotic scenario is feasible.^1^

A reaction with many products, all with similar production rates, can lead to an increase in the chemical diversity of the system with the potential for millions of molecules to all interact in a single environment.^25^ This is a situation some prebiotic chemists describe as chemical “mess”^13,26,27^ or “clutter”.^28^ This high chemical diversity can make it extremely difficult to detect and quantify compounds.^28^ As chemical diversity increases, so does chemical complexity, which we define as the number of reactions occurring in the system. Eventually, the chances of two specific molecules meeting to produce a desired intermediate become very low compared to the likelihood of them reacting with other components in the system. In this regime, productive chemistry becomes statistically impossible without either an ordering mechanism or the concentrating conditions found in prebiotically promising geochemical environments.

**Figure 1:**
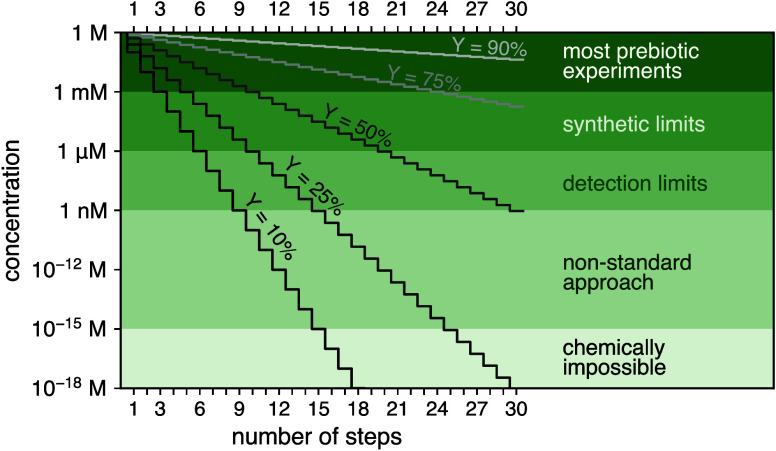
Concentration of a preferred product as a function of the number of steps in a synthetic chemical sequence. The shaded regions of different colors represent five different sequences where every reaction in each sequence has the same yield for the desired intermediate, ranging from 90% to 10%, with the yield labeled on the figure. The labels and colored regions indicate the chemical regimes described in the text.

An illustration of the arithmetic demon is given in Figure 1. This figure shows the reaction yields over a sequence of 30 reactions, and uses those yields to calculate the concentrations of the desired products at the end of a sequence of chemical reactions. To give some context to these numbers, we divide the range of concentrations into five labeled segments each spanning multiple orders of magnitude. The labels are meant to generally apply to this approximate range, and therefore exceptions to these labels may be found in the literature. The first label is “most prebiotic experiments”, indicating that prebiotic experiments that form the monomeric building blocks of life or their precursors tend to have products at concentrations of ≳10^–3^ mol/L (see Walton et al.^23^ and references therein). The limits for the products of a multistep synthesis to participate in subsequent reactions are around ≳10^–6^ mol/L.^23^ Most detection techniques will not operate reliably for concentrations below 10^–9^ mol/L^29^ meaning that most current or near-future prebiotic investigations will not be able to empirically test scenarios that involve concentrations much lower than this. Instead, nonstandard approaches, involving theoretical models or novel analytical techniques, will be required. There will be some concentration, not yet precisely determined, below which prebiotic synthesis is statistically impossible regardless of the yield.

Returning to the farming analogy, prebiotic synthetic chemists are those who seek to farm in uncultivated soil. At times, the task may seem daunting, akin to trying to grow crops in soil that has never been tended. However, one of the principles of do-nothing farming is that even uncultivated soil is still regulated by nature and can turn out to be more fertile than the farmer often imagines. Here the prebiotic chemist’s soil is liquid water. Liquid water is a very challenging solvent for organic chemistry due to its tendency to break down many prebiotically relevant compounds,^30^ including precursors to monomeric building blocks, such as hydrogen cyanide, other nitriles, and aldehydes. Many of these hydrolysis reactions are effectively irreversible.^31^ Water also poses a challenge for polymerization reactions involving these building blocks, as they require dehydration, a process that is thermodynamically disfavored in water.^32^ These challenges underscore why numerous prebiotic syntheses focus on eliminating water through techniques like freezing^33^ and drying.^34^

In almost every other domain of synthetic chemistry the problematic nature of water would not pose an issue as any efficient method to get from reactant to product is allowed. In synthetic chemistry there is a clear goal, and artificial intervention, if effective, is welcome. However, in prebiotic chemistry, this is not the case, as the chemistry that could have occurred on the early Earth must have been possible without the presence of a chemist. This is a regime where there remains a goal, life from nonlife, but where intervention to achieve such a goal *for its own sake* is implausible. At first glance, this situation is hard to imagine playing out in any way that does not lead to almost certain failure.^35^

It is here that we find inspiration from the Japanese farmer Masanobu Fukuoka. Fukuoka proposed a different approach to farming, which he called do-nothing farming.^36^ Fukuoka suggested first identifying what crops will grow best in what environments; second, finding ways to remove intervention from the farmer wherever possible; and third, identifying only those minimal interventions that cooperate with the natural course of the environment.

Regardless of the effectiveness of do-nothing farming in agriculture,^37^ the virtues of do-nothing prebiotic chemistry are clear. An approach that identifies natural environments in which a certain chemical sequence is likely to produce good yields, tests these reactions within such environments,^23^ and minimizes contingency,^19^ offers a straightforward method to remove the chemist. In essence, this approach serves as a bridge between laboratory experiments and sequences of events in geochemical environments while also estimating the likelihood of these events occurring in a specific order.

Aspects of this do-nothing approach have already been applied to prebiotic chemistry^14^ and have found surprising success. In fact, there are several instances where reduced control over the experimental setup, coupled with broader mimicking of “messier” geochemical conditions, has resulted in more successful chemistry, yielding the desired products or exhibiting the desired behavior. The use of initial chemical conditions informed by the geochemistry of a specific environment has resulted in chemical syntheses with higher yields and better selectivity. Notable examples include chemical pathways involving both sulfite^38−40^ and ferrocyanide.^39,41,42^ The integration of common mineral compositions and surface properties has led to new discoveries, such as the catalytic synthesis of amino acids from Krebs cycle intermediates,^43^ and the formation of enantiomerically pure RNA precursors from racemic mixtures.^44^ Mineral surfaces have also proven to be better templates for RNA polymerization when compared to artificial surfaces.^45^ Moreover, wet–dry cycles that do not completely dry, such as would occur at water-rock interfaces, turn out to be far more productive.^46^ Additionally, ultraviolet (UV) light can act to “tidy” the chemistry by selecting for nucleotides that are photostable and can be photochemically repaired; these turn out to be the nucleotides life uses.^47−49^ In addition, models suggest that phase equilibrium between gas-phase carbon and graphite can also simplify and order magma chemistry such that it looks astonishingly like laboratory reactant mixtures for prebiotic chemistry experiments.^50^ Based on these findings, it seems that in the right settings nature is far better at prebiotic chemistry than the trained organic chemist.

## Implementing the Do-Nothing Approach

Implementing the do-nothing approach involves identifying the most productive geochemical settings for prebiotic chemistry and determining what changes must still be imposed in order to mimic the unpredictable yet crucial natural events required for the emergence of life.

The first step when exploring any new chemical reaction is to determine the conditions required for the reaction to work. The next step is to figure out what products are formed and in what quantities. Based on this information we can make an estimate of the reaction yield and begin to ascertain if this is a steady-state yield, or whether it would change further if the reaction were given more time to progress. For some applications, this extent of investigation, along with some workup strategy, would be sufficient. For prebiotic chemistry, however, we want to translate the laboratory results to a natural environment.

The simplest and often first approach to applying laboratory results to natural environments is to measure reaction time scales. Here we define a time scale as the time taken for a given reaction sequence to build up steady state concentrations of desired products at a specified yield. Time scales measured in the lab will be determined by the physical conditions of the experiment. However, when applied to natural environments, the time scales will need to be adjusted to account for the specific conditions in the geochemical environment of interest. This is the approach taken by Rimmer et al. for UV-driven photochemistry.^1,2^ The utility of this approach is that it enables us to predict how a sequence of chemical reactions will play out in a natural environment. However, it is essential to acknowledge that these time scales will not exhibit straightforward relationships if the chemistry in the system is complex.

To translate these time scales from lab to environment, it is necessary to measure rate constants. These constants represent the capacity for a chemical species to react at a given rate.

For a reaction
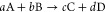
We can write out the rate equation for the concentration of C, [C], as
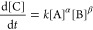
where *k* refers to the rate constant for the reaction, *t* is time, and α and β represent the order of reaction with respect to *A* and *B*. Given this representation, the experimental measurement of different species’ time scales can be related to the reaction rate. By varying the concentrations of these species we can reveal both the rate constant for this reaction and the order of reaction with respect to each reactant. The rate constant for a reaction is always context-dependent and can depend on factors such as temperature, pH, buffering capacity, and the flux of UV light. For example, Rimmer et al.^1^ measured the rate constant for the bimolecular formation of aminomethanedisulfonate as a function of temperature. When the context is broad, as is often the case for prebiotic chemistry, accurate measurement of rate constants can be laborious. However, this work is essential if we are to properly consider the environmental context for prebiotic chemistry.

To understand prebiotic chemistry in an environmental context, we will need to construct reaction networks. These networks provide a chemical map or picture of the environment. Systems prebiotic chemistry has already committed several years of very fruitful work to discovering reactions and constructing networks.^51^ In addition, the contrasting kinetic behaviors of living and nonliving systems can offer insights into the stepwise transition from nonlife to life.^12^ The measurement of rate constants has accompanied this endeavor for some time,^31^ but only recently has it gained significant momentum.^1,2,6,40−42^ Rates and time scales themselves are useful for evaluating prebiotic scenarios,^2^ however, rate constants allow for the application of models to assess the sensitivity of prebiotic scenarios to the environment.

**Figure 2:**
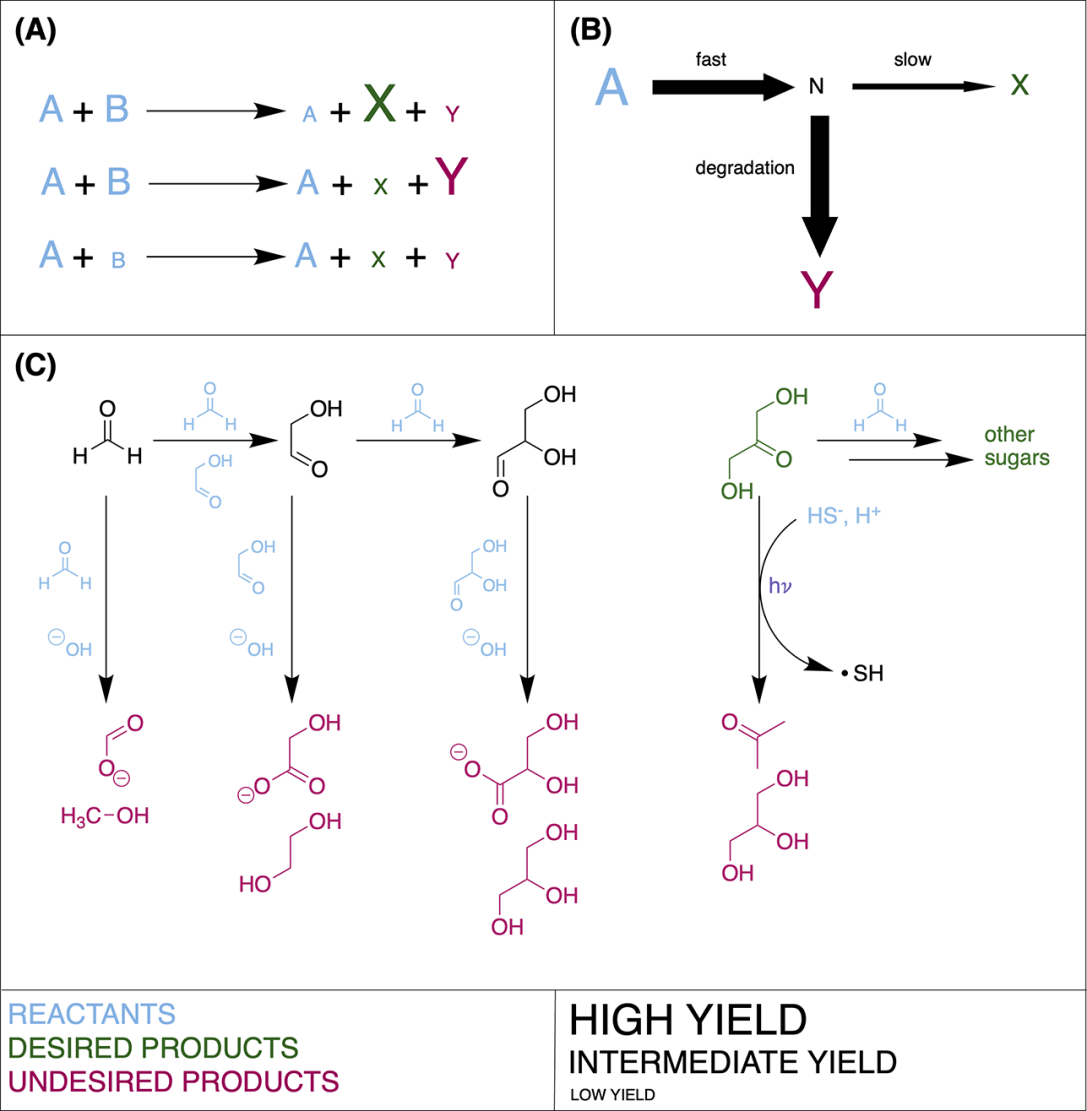
Different reactions in abstract (A, B) and a concrete example, the formose reaction (C). (A) shows a single reaction with reactants (blue), desired products (green) and undesired products (magenta). The bottom row of panels provide a key for the font color and size. The first reaction shown in (A) is a reaction with a high yield of a desired product, the second with a high yield of an undesired product, and the third with a low yield of both, due to the low concentration of the limiting reactant. (B) shows a sequence of reactions, where the yield of the intermediate, N, is low due to its short degradation lifetime (resulting in an undesired product) when compared to the time scale for the reaction that forms the desired product. (C) shows the formose reaction, which is autocatalytic in nature, with steps from formaldehyde to both end products and side products, as well as degradation products resulting from the interfering Cannizzaro reaction. In addition, we show one further reaction from the cyanosulfidic scenario, converting dihydroxyacetone to acetone and glycerin, a precursor for phospholipid synthesis.

Looking at reaction networks based on the kinetics of individual reactions allows us to estimate the yield and selectivity of a given pathway (see Figure 2(A,B)). In complex systems with many reactions occurring simultaneously, a subset of reactions that proceed significantly faster than others will dominate the system. Even if some side reactions are faster than those in the subset, the subset can still dominate if the reverse reactions are equally swift. As long as this subset of reactions dominates, the system can be described by a simpler network based on the subset. However, if the dominance changes, the network structure may shift, with other reactions becoming more prominent and some fading away. This underscores the necessity of using rate constants to identify the dominant reactions within a given environment. Determining these reactions is especially important when considering catalysis. A catalyst is a chemical species that increases the rate of a reaction without being consumed. A catalytic reaction is a reaction in which a catalyst is present at a concentration sufficient to measurably affect the rate. An autocatalytic reaction is a reaction where one of the products can act as a catalyst.

Catalysts are often sought by prebiotic chemists as they can promote desirable reactions. There are many examples of this in the literature; for instance, Kaur et al.^43^ From the synthetic chemistry perspective, catalysis helps enhance the yield and selectivity of a reaction. From a systems chemistry perspective, catalysts selectively enhance specific pathways within a network, thereby providing a degree of kinetic control. Autocatalytic reactions are of particular interest to the prebiotic chemist as they appear especially “life-like” in their behavior: for example, the replication of a strand of DNA is characterized by an autocatalytic set of reactions.^52^

These autocatalytic reaction networks are prevalent in many important prebiotic syntheses. For example, the formose reaction, shown in Figure 2(C), involves the formation of sugars, particularly aldose sugars like ribose, from the reaction of formaldehyde, or its derivatives, with glycolaldehyde.^53^ The formose reaction is described as autocatalytic due to the regeneration of glycolaldehyde.^54^ Although not fully understood it is thought that this reaction starts with the combination of glycolaldehyde and formaldehyde, producing glyceraldehyde. After further reactions glyceraldehyde then reacts with another molecule of formaldehyde producing compounds which eventually break down into two molecules of glycolaldehyde. These glycolaldehyde molecules can then re-enter the reaction.^55^ Despite the promising nature of this reaction scheme, the formose reaction is notoriously unstable,^20^ and the autocatalytic potential of this network can be difficult to manifest. The plausibility of this mechanism in a natural environment can be assessed by modeling its kinetics. If the rate of reaction for the combination of formaldehyde with glycolaldehyde is much faster than the combination of formaldehyde with the glyceraldehyde derivative, then the regeneration of glycolaldehyde would not be possible as all of the formaldehyde in the system would be used up. In addition, if Cannizzaro reactions are favored in an environment, the intermediates will all end up as alcohols and carboxylic acids (see Figure 2(C)). By studying the kinetics involved in each step of this reaction we can assess what reactions will dominate.

The formose reaction is just one example of an autocatalytic prebiotic reaction among many.^51^ Further research is necessary to ascertain the robustness of these autocatalytic reactions in natural environments, and other catalytic chemistry, including systems that exhibit mutual catalytic behavior,^56^ where they must contend with degradation chemistry. However, we propose that future studies may reveal these reactions to align with our definition of do-nothing prebiotic chemistry.

## Applicability of Do-Nothing Prebiotic Chemistry

When assessing the applicability of this do-nothing approach it is important to consider how we can effectively monitor our progress. Within origin of life research we focus on looking at the viability of different prebiotic scenarios. This involves scrutinising various combinations of proposed chemical pathways and environmental conditions. These scenarios aim to account for the transition from our initial chemical conditions to some end point that is, by some measure or judgment, closer to life as we know it.

There are many different ways this closeness to life, or “aliveness”,^12^ could be characterized, one of which is the level of self-control exhibited by an individualized chemical system. In life as we know it, an important component of this self-control is kinetic control, usually achieved through enzymes.^57^ The greater the extent to which a chemical system exerts control over itself and its immediate environment, the more we might consider it to have greater aliveness. Thus, kinetic control seems to be a necessary condition for life, though it is far from a sufficient condition.

Environments that structure the chemistry in such a way as to provide a measure of kinetic stability or redundancy for that system—by providing stable fluxes of feedstock molecules, naturally regulating rates so that the desired subset of reactions dominates, and storing and then releasing key chemical intermediates—are the environments that seem most apt to enable collections of molecules within them to self-organize. This serendipitous chemical ordering provided by the environment to the chemical system would only be coincidentally aimed toward the preservation and propagation of that collection of molecules. However, chemical systems directed toward survival and self-propagation will turn out to be the systems that have the greater chance of persisting.

We are not yet at a stage where we can fully identify the ideal combination of immediate environment and chemical system, however, we can identify certain global properties that the environment must possess for a specific prebiotic scenario. These include minimal ultraviolet spectral irradiance at the surface of a given rocky planet.

**Figure 3:**
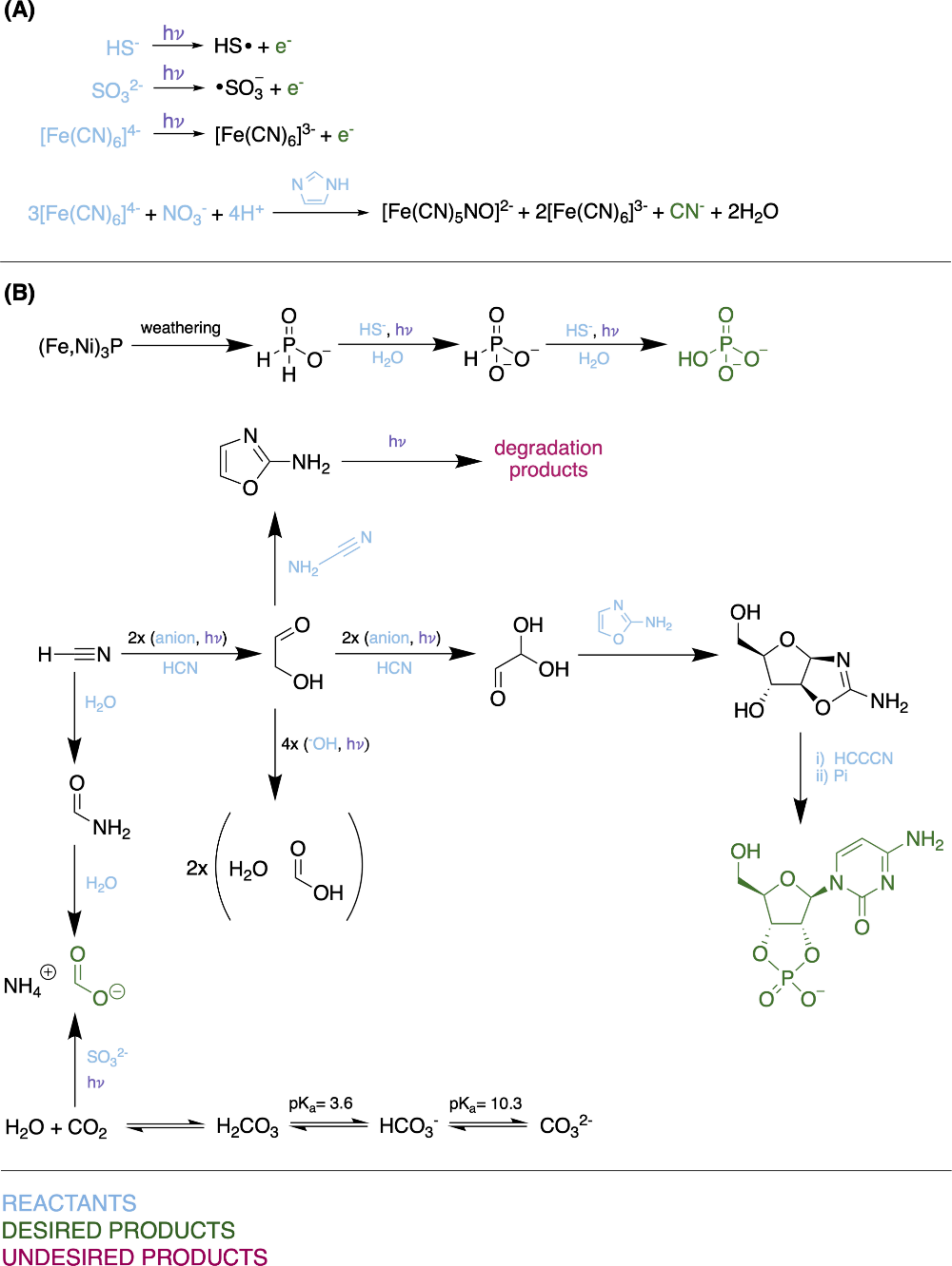
A simplified scheme for cyanosulfidic chemistry showing (A) the sources of solvated electrons and (B) the subsequent reduction of cyanide and various other species by those electrons, as well as the oxidation of hypophosphite by the leftover radicals. See Green et al.^9^ for a review of this chemistry.

**Figure 4:**
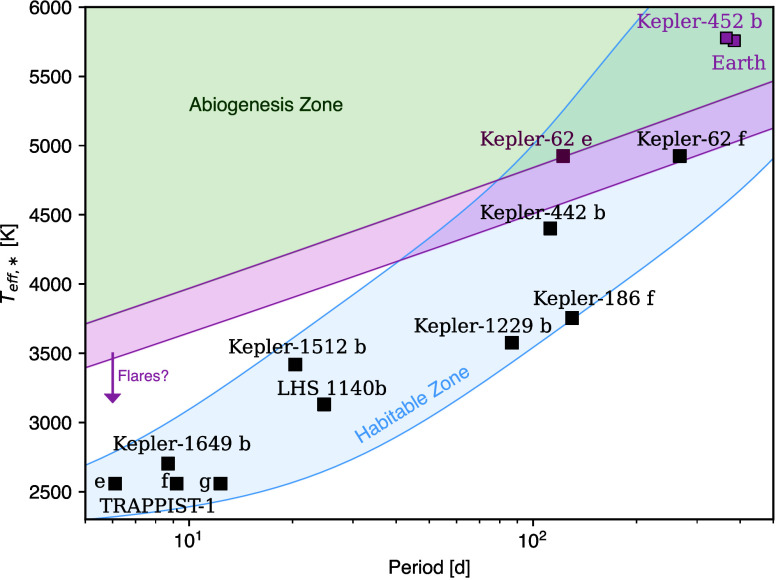
Exoplanets plotted by orbital period and the effective temperature of their host stars. Also shown is the delineation of both the liquid water habitable zone (blue region), where liquid water is predicted to be stable on the surfaces of rocky planets, and the abiogenesis zone (green region) with experimental error (magenta region), outside of which UV-driven cyanosulfidic chemistry is not possible. Figure taken from Rimmer et al.,^1^ their Figure 4, under Creative Commons.

Rimmer et al.^1^ applied kinetics and time scales to predict where UV-driven cyanosulfidic chemistry^58^ (see Figure 3) can take place. They investigated the production of simple sugars from hydrogen cyanide and sulfite, and from glycolonitrile and sulfide. At each of these steps, the chemistry can form inert adducts without UV light (dark chemistry) and precursors to sugars in the presence of UV light (light chemistry). In this work, they were able to determine a threshold UV spectral irradiance such that the light chemistry out-competes the dark chemistry, meeting the requirements of high yield and selectivity (see Figure 1). They then compared this threshold UV spectral irradiance to the UV spectral irradiance for various stars, at distances from those stars where the total flux of the star is equivalent to the flux received at the Earth from the Sun. This delineates an “Abiogenesis Zone” outside of which the cyanosulfidic scenario cannot succeed, if using sulfite as the source for solvated electrons. This zone is drawn out in Figure 4. These conclusions were drawn from applying kinetics to derive time scales appropriate for specific environments. Much more work has since been done to constrain the lifetimes of key prebiotic species, such as sulfite,^40^ ferrocyanide^41^ and aminoazoles.^6^ Alongside this, work has been done to constrain the kinetics of ferrocyanide formation,^42^ the pH-dependent rate constants for the photochemical production of formate from carbon species,^4^ and the time scales for various chemical steps in the cyanosulfidic network.^2^

**Figure 5:**
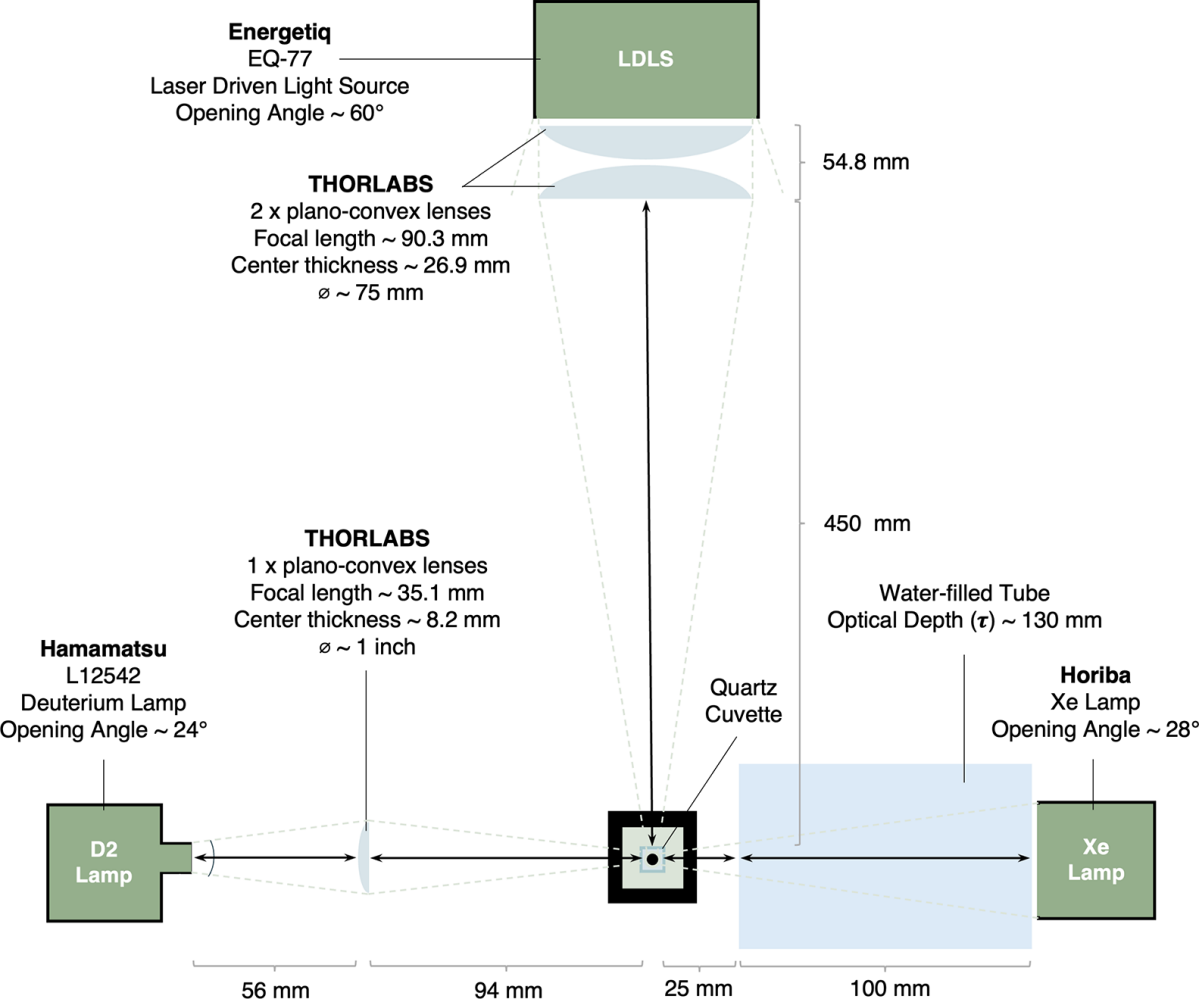
Irradiation Setup. All three light sources are shown and their opening angles indicated. Where applicable the lenses used and their specifications are shown on the diagram. We also show the distances between our sample holder and each lamp. The combination of these three sources can be used to reproduce broadband UV spectra of any shape.

Here we highlight three examples from our group, two of which are focused on exploring UV-driven prebiotic chemistry. For this we use an irradiation system as can be seen in Figure 5. This comprises of three light sources including a Laser Driven Light Source (LDLS) with a wavelength range from 200–800 nm that can be scaled to match the young Sun. Alongside this we also have both a D_2_ and a Xe Lamp which can irradiate the same sample in combination to reproduce broadband spectra of arbitrary shape, allowing us to simulate the broadband spectral irradiance, above 200 nm, of any star.

The first example relied on an earlier iteration of the irradiation setup to examine seven different reactions that comprise the cyanosulfidic network. These included the oxidation of phosphate, photodetachment of a hydroxide ion, reduction of cyanide via photodetachment of electrons from sulfite, photoanomerization of α-thiocytidine, photoreduction of thioanhydroadenosine and thioanhydrouridine, and nitroprusside synthesis from nitrite or nitrate in the presence of ultraviolet light.

The exploration of sulfite and iron photoredox chemistry cast our attention to Venus, where both iron and sulfur are present in abundance. We worked with the Tosca Lab in order to explore the production, lifetime, and ultraviolet absorbance properties of iron–sulfur minerals in the clouds of Venus, and discovered that one particular iron–sulfur mineral, predicted to be kinetically stable through the upper cloud layer, is a candidate for the unknown ultraviolet absorber observed in the clouds of Venus.^3^

More recently, we used an updated version of StarLab, as shown in Figure 5, to link the carboxysulfitic prebiotic reaction network, first proposed by Liu et al.,^59^ to specific geochemical environments. In this study, we determined the rate law for carbon reduction using solvated electrons, which are produced through electron photodetachment from sulfite, and measured the corresponding rate constants as a function of pH. Based on the observed rate of formate production, we suggested that this chemistry is most compatible with mildly alkaline aqueous environments exposed to UV radiation and in proximity to sources of both SO_2_ and CO_2_. Following kinetic calculations, we then determined approximate time scales for this chemistry on both the Archean Earth and early Mars.^4^

The three examples outlined here represent the first of many studies that will all have the goal of bridging the gap between experiments performed in the laboratory and the geochemical environment.

## The Future of Do-Nothing Prebiotic Chemistry

The goal of our lab is to find out the circumstances under which prebiotic chemistry can take place without a chemist. To do this we will need to construct prebiotic reaction networks and determine the kinetics encompassing the behavior of these chemical maps. A high fidelity picture of multiple prebiotic scenarios, at the level of chemical kinetics, will not be a task for a single group. Presently, we are constructing this picture, an aqueous photochemical kinetics model for prebiotic scenarios, in close collaboration with a small handful of others, however, such a monumental task will require the efforts of a community of dedicated scientists.

We suggest that integrating various chemical networks, including measured rate constants, within and outside our group, into this kinetics model, will facilitate the connection of ideas from numerous studies, thereby allowing a more comprehensive picture to emerge. As these models are slowly being constructed, it will be important to test the predictions of these models against experiments. Sensitivity analyses of these models will help identify those reactions and associated rate constants that most strongly determine the chemistry, emphasizing the importance of reliable experimental determination of these rate constants in particular.

These models are nonlinear: they are described by a set of deterministic ordinary differential equations that depend on powers of concentrations. Therefore, the time-evolution of the system described by these equations will be highly sensitive to the system’s initial conditions and parameters. The set of equations is determined by the chemical network, however, the network employed is more an expression of our own knowledge of the system than a complete representation of reality. Determining when a network is comprehensive enough to accurately represent the system of interest is a challenging task. How can one determine if a key reaction essential for characterizing a system has been overlooked, or if it has been assigned an incorrect mechanism? Additionally, how can it be verified whether a proposed reaction, deemed critical for the predicted behavior, is in fact occurring? Due to the inherently nonlinear nature of these models, the complexity of the networks, and the poorly understood criteria for the “completeness” of a network, there is a risk that the system will deviate from experimental observations. This is why it will be essential for multiple models to be independently constructed and tested against experimental and observational data, such as data from laboratory simulations of planetary environments and observations of those environments, especially observations from Mars sample return missions, where the geological history is well-preserved when compared to Earth.

While we suggest that this approach is ideal for exploring and testing the first stages of prebiotic chemistry, we acknowledge that eventually the level of complexity will exceed what can be practically modeled using chemical kinetics modeling. Just as it would be inappropriate to model planetary formation using the tools of quantum field theory, the chemical complexity at the interface of the transition from nonlife to life will require a different set of tools appropriate to this new explanatory regime. The discovery of this point of transition by pressing on the boundaries of what chemical kinetics is capable of would itself qualify as a significant breakthrough into a new phase of research into life’s origins.

## Conclusion

In this account we presented our motivation to understand how prebiotic chemistry could happen without a prebiotic chemist, an approach we call do-nothing prebiotic chemistry.

There is a chasm, of orders of magnitude, spanning both space and time that separates the chemist from the chemistry, yet in the natural environment, this distinction fades. The ever-evolving landscape granted by nature may be a better host for the chemistry than the laboratory, its design limited by such practical considerations as the time it takes to finish an experiment, analytical limitations, and safety. Nature is ever changing, however, its laws and rules are always and everywhere the same, and so the chemistry of a natural environment will inevitably unfold according to the rules of chemical kinetics, rules that can be measured in the lab. In this way, the “Frankenstein” constructed in the lab,^60^ may pave the way for discovering the emergence of life on a young planet, so long as the chemical kinetics unfold as expected. As a result, kinetics can act as a bridge between these two regimes.

This bridge between laboratory and geochemical environment is far from built. Here we have provided a brief review of the initial components of scaffolding we and other groups have put up. Our work will need continued support for us to make any real progress. Without this collaboration, we may never uncover how life could have emerged in a natural environment, as the chemistry cannot be applied beyond the confines of the laboratory. While we point out that not all groups need to measure rate constants, we emphasize the importance of considering these measurements when conducting experiments and communicating results.
